# Disruption in the Regulation of Immune Responses in the Placental Subtype of Preeclampsia

**DOI:** 10.3389/fimmu.2018.01659

**Published:** 2018-07-20

**Authors:** Janri Geldenhuys, Theresa Marie Rossouw, Hendrik Andries Lombaard, Marthie Magdaleen Ehlers, Marleen Magdalena Kock

**Affiliations:** ^1^Department of Medical Microbiology, Faculty of Health Sciences, University of Pretoria, Pretoria, South Africa; ^2^Department of Immunology, Institute for Cellular and Molecular Medicine, University of Pretoria, Pretoria, South Africa; ^3^Obstetrics and Gynecology, Rahima Moosa Mother and Child Hospital, Wits Obstetrics and Gynecology Clinical Research Division, Faculty of Health Sciences, School of Clinical Medicine, University of Witwatersrand, Johannesburg, South Africa; ^4^Department of Medical Microbiology, Tshwane Academic Division, National Health Laboratory Service, Pretoria, South Africa

**Keywords:** preeclampsia, trophoblast invasion, immune regulation, pregnancy, monocytes, inflammation

## Abstract

Preeclampsia is a pregnancy-specific disorder, of which one of its major subtypes, the placental subtype is considered a response to an ischemic placental environment, impacting fetal growth and pregnancy outcome. Inflammatory immune responses have been linked to metabolic and inflammatory disorders as well as reproductive failures. In healthy pregnancy, immune regulatory mechanisms prevent excessive systemic inflammation. However, in preeclampsia, the regulation of immune responses is disrupted as a result of aberrant activation of innate immune cells and imbalanced differentiation of T-helper cell subsets creating a cytotoxic environment *in utero*. Recognition events that facilitate immune interaction between maternal decidual T cells, NK cells, and cytotrophoblasts are considered an indirect cause of the incomplete remodeling of spiral arteries in preeclampsia. The mechanisms involved include the activation of immune cells and the subsequent secretion of cytokines and placental growth factors affecting trophoblast invasion, angiogenesis, and eventually placentation. In this review, we focus on the role of excessive systemic inflammation as the result of a dysregulated immune system in the development of preeclampsia. These include insufficient control of inflammation, failure of tolerance toward paternal antigens at the fetal–maternal interface, and subsequent over- or insufficient activation of immune mediators. It is also possible that external stimuli, such as bacterial endotoxin, may contribute to the excessive systemic inflammation in preeclampsia by stimulating the release of pro-inflammatory cytokines. In conclusion, a disrupted immune system might be a predisposing factor or result of placental oxidative stress or excessive inflammation in preeclampsia. Preeclampsia can thus be considered a hyperinflammatory state associated with defective regulation of the immune system proposed as a key element in the pathological events of the placental subtype of this disorder.

## Introduction

Preeclampsia is a multisystem heterogeneous disorder unique to pregnancy and is considered a leading cause of maternal as well as fetal/neonatal morbidity and mortality (1). The World Health Organization has estimated 50,000–60,000 preeclampsia-related maternal deaths per year worldwide (2–4). Preeclampsia is a syndrome defined as *de novo* gestational hypertension [systolic- and diastolic blood pressure (BP) of more than 140/90 mmHg respectively] plus new-onset proteinuria (minimal of 300 mg), or one or more signs of systemic involvement (5, 6). The clinical spectrum includes manifestations of the maternal and fetal component associated with reduced blood flow and abnormal oxygenation in the placenta, intrauterine growth restriction (IUGR), and prematurity (7, 8). Adverse clinical conditions and maternal organ dysfunction associated with preeclampsia include renal insufficiency, liver involvement, neurological or hematological complications, and uteroplacental dysfunction (9). These conditions can progress to eclampsia, stroke, uncontrolled severe hypertension, acute kidney injury, liver hematoma, liver rupture, and cardiac failure as well as severe complications also involving the fetus by possible abruption of placental membranes and stillbirth (5). A 12-fold increase in the risk of cardiovascular disease has been found in women with a history of preeclampsia and metabolic disease, highlighting a relationship between preeclampsia and cardiovascular disease (10).

Preeclampsia is characterized by two major subtypes: the maternal subtype also known as the metabolic immunologic subtype and the placental subtype that entails placental ischemic–hypoxic stress followed by systemic maternal inflammation. Although immune dysregulation plays a substantial role in both subtypes, the two subtypes have different etiologies and phenotypes, while the placental subtype refers to early-onset preeclampsia with an etiology of abnormal placentation under hypoxic conditions (11). The pathogenesis of preeclampsia was originally ascribed to endothelial dysfunction (12), which also plays a central role in the development of cardiovascular disease. In fact, preeclampsia shares many risk factors with cardiovascular disease, such as obesity, hypertension, insulin resistance, and dyslipidemia, all conditions, which are characterized by inflammation (13–16). It is now believed that adverse immune responses generate the endothelial dysfunction that can lead to hypertension in pregnant women (17). Pregnancy already imposes an immunological challenge on the host, since direct contact of circulating and uterine immune cells with placental tissue requires adaptations by the maternal immune system to maintain tolerance to the fetus (18). The exact pathogenesis of preeclampsia is, however, still unclear and has resulted in multiple hypotheses about the underlying mechanisms (19). One such hypothesis is that the etiology of preeclampsia is primarily immunological, since immune mechanisms are the interconnection between placental ischemia and maternal cardiovascular disease (17, 20). The placenta is a major etiological factor in the pathogenesis of preeclampsia and other etiological factors such as placental cells, angiogenic and antiangiogenic proteins involved in the complex pathology of preeclampsia are described later in the widely accepted two-stage model. Briefly, poor placentation results in an oxidatively damaged placenta (21, 22) that releases several placental factors into the maternal circulation, eliciting a maternal systemic inflammatory response and endothelial dysfunction (23). This review briefly discusses the aspects associated with the placental subtype of preeclampsia with special focus on the dysregulation of immune responses.

## The Pathological Events of Preeclampsia

The placental cells involved in the pathological events of preeclampsia are specialized extravillous cytotrophoblasts and villous syncytiotrophoblasts, which have distinctive proliferative and invasive properties (19). These separate subgroups originate from two different villous cytotrophoblast precursors (24). Extravillous cytotrophoblasts are differentiated into an invasive phenotype, with high migratory, proliferative, and invasive properties (25). During weeks 8–18 of normal pregnancy, cytotrophoblasts invade the decidua to induce extensive remodeling of the uteroplacental spiral arteries (21). Remodeling of these spiral arteries is important for reducing resistance to maternal blood flow to enable efficient blood supply to the fetal compartment (26). This process is called placentation and effectively modifies the quality of maternal blood flow to be non-pulsatile and ensures a low-pressure state in the placenta (21). An optimal uterine environment is established to meet the metabolic demands and the required rate of the physiological exchange of nutrients and oxygen between the maternal and fetal systems (27). In the initial stage of preeclampsia, cytotrophoblasts fail to invade the decidua and restrict the subsequent modification of the uteroplacental spiral arteries (8). However, poor placentation is not the only cause of the placental subtype of preeclampsia, but acts as a predisposing factor to the development of a maternal syndrome with immunological involvement (7, 21). The complex pathology of preeclampsia can be explained according to a widely accepted two-stage model (21).

### The Pathological Events of Preeclampsia in a Two-Stage Model

The pathophysiology of preeclampsia has been formalized in a two-stage model that delineates the evolution from poor placentation to the maternal clinical syndrome (22). As described above, poor placentation is due to the incomplete remodeling of the spiral arteries, resulting in high BP flow and irregular delivery of fully oxygenated arterial blood to the placenta (21). The latter is termed placental ischemia/hypoxia and is associated with distortion of the placental villous architecture and increased oxidative stress (21). The subsequent uteroplacental insufficiency results in compromised blood flow to the uterus, resulting in pregnancies with increased perinatal morbidity and mortality (1). However, incomplete remodeling of the spiral arteries is not only associated with preeclampsia but is also found in IUGR and gestational hypertension (22, 28), suggesting that it is not the only cause of preeclampsia. During the initial stage of preeclampsia, poor placentation occurs with no clinical features, followed by a second stage that involves a maternal clinical syndrome with cardiovascular manifestations and renal features (17, 21, 29).

Inadequate invasion of cytotrophoblasts following the incomplete remodeling of spiral arteries exposes the placenta to oxidative stress (22). The second stage of the disorder ensues because the ischemic placenta and increased placental oxidative stress cause excessive systemic inflammation and endothelial dysfunction that manifest as new-onset hypertension and proteinuria (21, 22, 29, 30). The end result of oxidative stress in preeclampsia is exaggerated placental necrosis or apoptosis, which are common histologic features of the preeclamptic placenta (21, 31). Excessive shedding of syncytiotrophoblast microparticles, reflecting placental ischemia and apoptosis of placental cells, into the maternal circulation, creates an inflammatory burden and indirectly affects endothelial function (32–35). This role of a diseased placenta in preeclampsia is part of the emerging theory classifying preeclampsia as two different diseases, either placentogenic or maternogenic (36). The different phenotypes of preeclampsia can thus either be placentogenic, which usually occurs in early pregnancy with association of poor placentation and different severities of IUGR, or maternogenic, which occurs late in pregnancy with no relation to placental insufficiency or IUGR (37). The metabolic syndrome (defined as the occurrence of hypertension, ischemic heart disease, type 2 diabetes mellitus, obesity, and insulin resistance) has also been associated with increased circulating microparticles and subsequent systemic inflammation (38).

The exact stimuli inducing inflammation in preeclamptic patients are, however, incompletely understood and various other possibilities exist (32, 39). For instance, an injured endothelium may release several factors thought to play a role in this systemic inflammatory response (40). These factors include pro-inflammatory cytokines, markers of oxidative stress, thrombomodulin, fibronectin, endothelin-1, and Von Willebrand factor (40, 41). Other placental factors, such as anti-angiogenic [soluble fms-like tyrosine kinase (sFlt-1)] factors secreted by placental cells can also be considered as possible stimuli, since these factors affect the endothelium, a component of the inflammatory system (22, 29, 34). Protein toxic aggregates such as aggregated transthyretin which, is a placental toxin elevated in preeclampsia, have been suggested to contribute to the pathogenesis of this disorder. Elevated levels of aggregated transthyretin are formed in preeclampsia and transported through the secretion of placental extracellular vesicles by syncytiotrophoblasts (42). Aggregated transthyretin in these vesicles may also allow targeted delivery of these toxic proteins to other maternal organs, conducting a signal of cellular stress from the diseased placenta and contributing to the pathogenesis of preeclampsia (42). Protein toxic aggregates is a novel approach, they have been considered to be pathogenic in other diseases, thus the possibility of contributing to excessive inflammation in preeclampsia through creating cellular stress should also be considered (42–44).

The role of inflammatory markers with elevated levels in preeclampsia has also been investigated, including the role of procalcitonin (PCT) and C-reactive protein (CRP) (45). PCT is a precursor of calcitonin and is used as a marker of bacterial infection and resultant systemic inflammation (45, 46). The exact role of PCT and CRP in preeclampsia remains controversial, with the literature revealing conflicting accounts of their usefulness in predicting preeclampsia and its severity (45).

It has been well established that the human microbiota plays a fundamental role in the functioning of the immune system and the regulation of immune responses (47). Microbiota–immune interaction is mediated through microbial-associated molecular patterns; for instance, the bacterial endotoxin lipopolysaccharide (LPS) is recognized by cellular toll-like receptors (TLRs) (47). Researchers have established LPS to be an external stimulus, which is able to induce hypertension and proteinuria identical to preeclampsia in pregnant rats (48). In this particular animal model of human preeclampsia, lipid A/LPS, a known pro-inflammatory stimulus, provoked systemic inflammation and was associated with the clinical manifestations of preeclampsia (48). In a study by Cotechini et al. (49) performed on pregnant rats, the administration of LPS induced a systemic and local inflammatory response, fetal growth restriction (FGR), and an increase in mean arterial pressure, through a mechanism mediated by tumor necrosis factor (TNF). In addition, the authors observed deficient trophoblast invasion and impaired spiral artery remodeling characteristic of preeclampsia, to be linked to LPS-induced FGR (49). Similar results were found by Xue et al. (50) who demonstrated that a single administration of LPS to pregnant rats induced inflammation specifically by binding to its receptor, the TLR-4 in the placenta. TLR-4 signaling in the placenta was associated with deficient trophoblast invasion and spiral artery remodeling contributing to poor placentation that may result in a preeclampsia-like syndrome (50). Ultimately, all of these secreted placental factors and stimuli, including bacterial endotoxin, are capable of disrupting the fine-tuned balance of the immune system and inducing a systemic inflammatory response.

### Angiogenesis as Part of Placentation and Biomarkers Present in Preeclampsia

**Figure 1:**
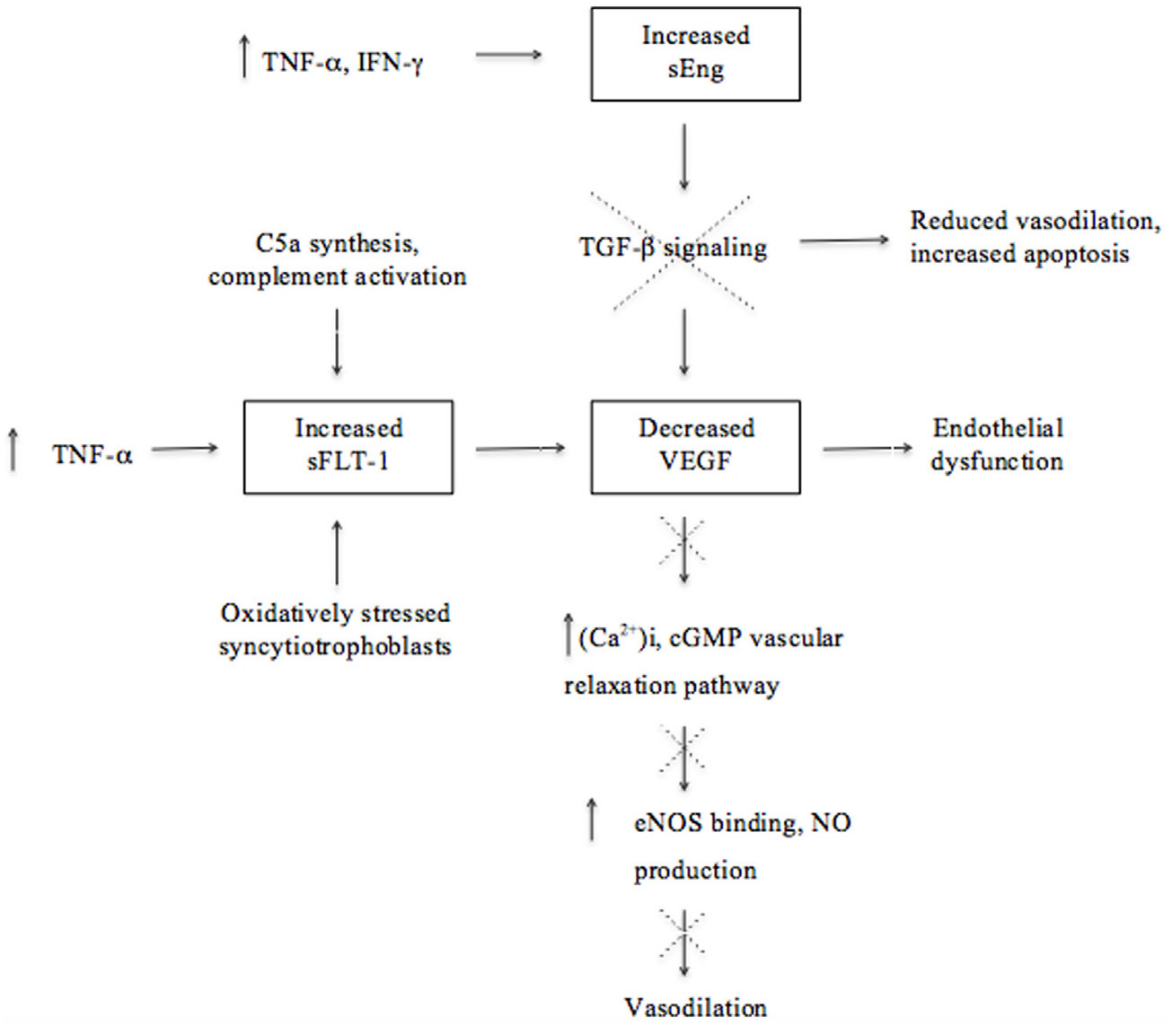
Molecular mechanisms involved in the upregulation and downregulation of antiangiogenic and angiogenic factors ultimately leading to endothelial dysfunction and reduced vasodilation in the pathogenesis of preeclampsia. Although there is a lack of proof that soluble fms-like tyrosine kinase (sFlt-1) is associated with excessive systemic inflammation, this illustration emphasizes the effect of the immune system on angiogenesis and that dysregulation thereof could contribute to the pathogenesis of preeclampsia.

The extensive angiogenesis that is characteristic of successful placentation is indirectly disrupted by placental ischemia/hypoxia in preeclampsia (1, 27, 51). Angiogenesis is critical for the improvement of placental circulation and blood flow by the formation of new vascular beds to enable vascular growth and development of the placenta (27). Placental factors, specifically pro-angiogenic [vascular endothelial growth factor (VEGF) and placental growth factor (PIGF)] and anti-angiogenic proteins, are responsible for placental growth, vascularization, and maintenance of vessel health (8, 27). These factors act as biomarkers that reflect unique patterns in preeclampsia according to the severity as well as the gestational age at the onset of the disorder (52). Preeclampsia is associated with decreased levels of vasodilators, such as nitric oxide (NO) and prostacyclin, as a result of the disproportionate increase in sFlt-1 and decrease in VEGF (Figure 1) and PIGF (53–55).

The growth factor VEGF and its homolog, PIGF, are essential for angiogenesis because of their pro-angiogenic activity that induces vascular permeability and promotes the proliferation and survival of epithelial cells (40). VEGF may cause vasodilation by stimulating the NO-cyclic guanosine monophosphate vascular relaxation pathway as well as by increasing the production of the calcium ion (Ca^2+^)i (53). An increase in (Ca^2+^)i promotes the binding to endothelial NO (eNOS) and subsequently increases eNOS activity and stimulates NO production (Figure 1) (53). Transforming growth factor (TGF)-β1 is involved in angiogenesis by regulating the expression of VEGF by means of intracellular signaling (40, 56). The inhibition of TGF-β1 signaling will lead to reduced endothelium-dependent vasodilation and increased endothelial cell apoptosis, suggesting that dysregulated TGF-β1 signaling may be involved in the pathogenesis of preeclampsia (40). A decline of VEGF results in glomerular endotheliosis, a specific endothelial renal lesion presenting with proteinuria (21). In addition to low VEGF concentrations, a decline in NO, PIGF, and TGF-β1 levels, in concert with an increase of anti-angiogenic factors, deprives endothelial cells of support leading to endothelial dysfunction (8, 17, 22). Thus, endothelial dysfunction is a result of an antiangiogenic state in the placenta and contributes to the pathology of preeclampsia (Figure 1) (9, 52, 57).

Immunological factors also play a role in the secretion of antiangiogenic factors. For instance, complement activation has been shown to stimulate monocytes to release antiangiogenic factors (20). Studies in murine models of experimental pregnancy loss demonstrated that conditions of hypoxia and inflammation lead to the release of increased amounts of antiangiogenic factors and are therefore strong triggers of angiogenic dysregulation (20, 58). These studies and those in humans demonstrate that pregnancy complications, such as recurrent pregnancy loss (RPL), preterm birth, and preeclampsia, are associated with excessive complement activation, especially enhanced C5a synthesis, that promotes the secretion of the antiangiogenic factor sFlt-1 (20, 59). Activation of C5 contributes to fetal loss and adverse pregnancy outcomes by inducing a dysregulation of angiogenic factors and initiating a complex series of events, as demonstrated in the abortion-prone mouse mating combination CBA/J and DBA/2 (20). The importance of activation of complement through the lectin pathway was further illustrated with the administration of inhibitory factors, such as Polyman2, which neutralizes mannose-binding lectins and anti-C5, a neutralizing recombinant antibody that prevented fetal loss in this mating combination (20, 60). The important role of disruption in the regulation of complement activation in preeclampsia is also demonstrated by the findings of C4A deficiencies and C4b deposits in preeclamptic placentas (61, 62).

Soluble fms-like tyrosine kinase (also referred to as sVEGFR-1) is a soluble form of the VEGF/PIGF receptor Flt-1 and is a potent inhibitor of VEGF and PIGF angiogenic activity (51, 53–55). Elevated levels of sFlt-1 are associated with preeclampsia and have been found to induce hypertension, proteinuria, and glomerular capillary endotheliosis when administered to pregnant animals (63). Increased levels of sFlt-1 are more pronounced at an early stage before the onset of preeclampsia and are associated with several associated risk factors, including diabetes mellitus and gestational hypertension (52, 64, 65). Excess levels of sFlt-1 inhibit cytotrophoblast differentiation and invasion and subsequently contribute to poor placentation (20). A chronic increase in the production of pro-inflammatory cytokine, TNF-α, was found to induce hypertension in rats and stimulate the excessive secretion of sFlt-1, further supporting the effect of the immune system on inadequate angiogenesis in preeclampsia (17). Excessive secretion of sFlt-1 is also suggested to be induced by hypoxic conditions and reflect the response of oxidatively stressed syncytiotrophoblasts during poor placentation. Therefore, possible sources contributing to the higher concentrations of sFlt-1 observed during preeclampsia are the syncytiotrophoblasts in the placenta in the presence of oxidative stress, as well as peripheral blood mononuclear cells, macrophages, and endothelial- and vascular smooth muscle cells (34, 66).

Increased concentrations of another antiangiogenic factor, soluble endoglin (sEng), in preeclampsia contributes to endothelial dysfunction through its inhibition of TGF-β1 signaling (35, 67). Elevated levels of sEng could be attributed to oxidative stress and hypoxic conditions in the placenta as well as to stimulation by inflammatory cytokines, such as TNF-α and interferon (IFN)-γ, from endothelial or placental cells during non-hypoxic conditions (29, 68). The combined action of increased sFlt-1 and sEng concentrations is suggested to characterize preeclampsia better than a single analyte, emphasizing the role of several antiangiogenic factors in clinical preeclampsia (40, 69). Considering that the endothelium is a component of the systemic inflammatory system, elevated levels of antiangiogenic factors (sFlt-1 and sEng) secreted by a diseased placenta may contribute to systemic inflammation (Figure 1) (29).

In contrast to the pattern of the antiangiogenic factors, pro-angiogenic PIGF and VEGF are present in decreased concentrations in preeclampsia even before clinical manifestation occurs (52). The patterns of these biomarkers are considered relevant and an altered ratio of pro-angiogenic to anti-angiogenic factors is correlated with the severity of the disorder (70). Increased concentrations of sFlt-1 lead to PIGF and VEGF being sequestered (35, 52); hence, an imbalance in the expression of angiogenic and antiangiogenic factors leads to inadequate angiogenesis and disruption of the maternal endothelium and immunological balance advancing to the clinical signs of preeclampsia (Figure 1) (9, 17, 52).

### Stimulation of the Immune System at the Fetal–Maternal Interface Through the Recognition of Antigens

The systemic inflammatory response associated with preeclampsia involves immunological conflict originating in the decidua where maternal immune cells are in close contact with trophoblasts at the feto-maternal interface operative in placental tissue and the secondary (peripheral) lymphoid organs (28, 29, 34, 71, 72). In pregnancy, the maternal immune system is immunologically naïve to the tissue-specific antigens from the fetus and placenta as well as foreign antigens from the paternal genome (72). The maternal immune system relies on immune regulatory mechanisms to avoid rejection of the semi-allogeneic fetus while protecting the host against infections (73). Primary contact with maternal immune cells occurs as cytotrophoblasts invade the decidua (34, 71). Close contact between cytotrophoblasts and decidual immune cells will influence maternal immune cells to either cause tolerance, and subsequently facilitate trophoblast invasion, or elimination at the feto-maternal interfaces (28, 34, 74, 75). In addition, the syncytiotrophoblast is in contact with immune cells in maternal blood flowing through the intervillous space (71, 76). This contact can be direct or indirect through the release of soluble factors (e.g., cytokines), immunosuppressants (e.g., progesterone and prostaglandins), specific suppressor molecules [e.g., soluble isoforms of human leukocyte antigen (HLA)-G and HLA-E], tolerogenic molecules [e.g., TGF-β1 and interleukin (IL)-10], and immunomodulatory products [e.g., indoleamine 2,3-dioxygenase (IDO), Fas ligand (FasL), and TNF-related apoptosis-inducing ligand (TRAIL)] (71, 72, 77).

Paternally derived antigens are expressed on trophoblasts and recognition events by the maternal immune system stimulate an immune response from innate and adaptive immune cells, which controls trophoblast invasion and placentation through the release of trophic substances such as cytokines and PIGFs (21). Interaction of cytotrophoblasts with target cells facilitates maternal–fetal immune recognition through receptors that recognize HLA ligands (78, 79). The classes of HLA antigens differ in their preference for specific immune cell interactions to facilitate their important role in immune regulation (71). In humans, unique combinations of the genotypes of class I major histocompatibility complex (MHC) antigens, the classic (polymorphic) HLA-C and non-classic (non-polymorphic) HLA-G are expressed on extravillous cytotrophoblasts (71). The lack of expression of the highly polymorphic classical MHC class I antigens (HLA-A and -B), the principal stimulators of graft rejection, and class II antigens is an important mechanism to escape immune rejection of the fetus (71, 80).

Human leukocyte antigen C is recognized by decidual T cells and decidually derived CD16^−^CD56^bright^ natural killer (dNK) cells (79, 81). These dNK cells are phenotypically and functionally distinct from NK cells in the peripheral circulation and have unique functions, including the production of chemokines, cytokines, and growth factors, in addition to reduced cytotoxicity; qualities that make their presence acceptable at the feto-maternal interfaces (81, 82). These functional differences may indicate a distinct NK cell lineage, or alternatively, that these changes are induced by the unique decidual microenvironment that is characterized by physiological stress hypoxia in the placenta and regulation by estrogen, progesterone, and trophoblast-derived soluble factors, such as soluble HLA-G (81, 82). While the origin of dNK cells is still unclear, Carlino et al. (83) have suggested that these cells are recruited from peripheral NK cells able to migrate through endothelial and stromal decidual cells, partly due to its higher migratory ability during the first trimester of pregnancy (83). Once peripheral NK cells come into contact with stromal cells, they acquire a chemokine receptor profile similar to that of dNK cells, indicating that this communication between NK cells modulated the NK cell phenotype (83). Decidual NK and T-cell subsets constitutively express killer immunoglobulin-like receptors (KIRs) at the fetal–maternal interface, composed of a series of inhibitory and activating receptors that interact with HLA ligands (84). Polymorphic KIRs, consisting of haplotype A and B, are distinguished based on their activating or inhibitory properties (28, 76).

The maternal KIR B haplotype has between one and five activating receptors while the KIR A haplotype contains only inhibitory receptors (85). Decidual NK cells display a specific KIR repertoire that is biased toward recognizing HLA-C, the dominant KIR ligand found on fetal extravillous trophoblasts (86). The specific ligand–receptor interaction of KIR BB and HLA-C ligand genotypes favors proper activation of dNK cells for the production of immunoregulatory cytokines and angiogenic factors (PIGF and VEGF, TGF-β1, and angiopoietin 1 and 2) that promote placental development (25, 81, 87–89). Adequate activation of dNK cells will thereby supply sufficient dNK-derived growth factors and chemokines to allow for adequate trophoblast invasion and vascular remodeling (81). Generally, weaker inhibitory interactions are associated with better protection against viral infection, or greater susceptibility to autoimmune conditions, secondary to greater NK cell activation (90). The HLA-G interacts best with KIR2DL4 receptors on dNK cells to stimulate the production of angiogenic factors as well as pro-inflammatory and immunoregulatory cytokines (71, 91, 92). Soluble HLA-G may also contribute to immune tolerance by upregulating immunosuppressive NK subsets (84). Although the principal interaction of these antigens is with dNK cells, HLA-G also interacts with the CD8 receptor on T cells. The latter interaction subsequently activates the Fas/FasL pathway, promoting the apoptosis of activated CD8^+^ T cells, and protecting trophoblasts from a T cell-mediated attack (71, 92).

Immune interaction with KIRs resulting in too strong inhibition prevents NK cell activation and this is postulated to play a role in preeclampsia where NK cell activation is needed to stimulate vascularization (90). Some have shown that insufficient activation of dNK cells contribute to the development of preeclampsia by promoting the lysis of trophoblast cells lacking HLA-G and the loss of trophoblast cells that should invade the developing spiral arteries, resulting in the insufficient supply of oxygen and nutrients to a developing placenta (93). Therefore, dNK cells stop the process of placental development and spiral artery remodeling by the extravillous cytotrophoblasts (93, 94). Protection against the development of preeclampsia therefore relies on adequate activation of dNK cells by receptor–ligand interactions that favor activating KIR receptors (haplotype BB) above ligation to inhibitory receptors (haplotype AA) (23, 81, 95). When maternal activating receptors are, however, absent as in the KIR AA genotype, binding to fetal HLA-C2 increases susceptibility to preeclampsia (79, 85). In preeclampsia, the activation of inhibitory KIR receptors through HLA-C2 binding is associated with inadequate immune recognition and trophoblast invasion (28, 71, 75). Altered dNK cell activation also results in inadequate angiogenesis. For instance, KIR-AA and HLA-C receptor–ligand interaction is responsible for the defective secretion of angiogenic factors by dNK cells and the increased expression of antiangiogenic factors, sFlt-1 and sEng, previously described to cause endothelial dysfunction (59, 87, 96).

Reduced levels of expressed HLA-G and its soluble concentration in maternal plasma were observed in cases of miscarriage and preeclampsia, suggesting that inadequate immune recognition and tolerance contributed to pregnancy complications in these women (97, 98). In addition, preeclamptic pregnancies have reduced levels of progesterone along with reduced levels of immunomodulators, such as IDO and TRAIL, and soluble CD30, a member of the tumor necrosis superfamily of receptors and a marker of T-helper (Th)2 polarization (99–101). These findings, in addition to those of epidemiological studies, support the hypothesis that immune maladaptation play a role in the pathophysiology of preeclampsia by means of an inappropriate release of cytokines and placental factors that cause both shallow invasion of trophoblasts and endothelial dysfunction (102, 103).

## The Key Role Players in the Disruption of the Immune System in Preeclampsia

The innate and adaptive components of the immune system play an important role in immune regulation to ensure a successful pregnancy (104). Various immune cells most notably dNK cells, decidual macrophages, T cells, and dendritic cells (DCs) are involved in this process (105). Decidual NK cells, the most abundant decidual leukocytes in the first trimester, are only weakly cytotoxic and are an important source of immunoregulatory cytokines, matrix metalloproteinases (MMPs), and angiogenic factors that promote key processes in placentation, such as extracellular matrix remodeling, trophoblast invasion, and angiogenesis (106–108). Decidual macrophages are present throughout pregnancy and similarly produce factors associated with tissue remodeling and angiogenesis, e.g., MMP9 and VEGF (109). These macrophages are mostly of the immunomodulatory phenotype (M2) and contribute to the creation of a tolerogenic immune environment by producing immunosuppressive cytokines, such as IL-10 and IL-35, inducing expression of regulatory T cells (Tregs), phagocytosing apoptotic trophoblast cells to prevent the release of pro-inflammatory substances, and inhibiting the cytotoxic function of dNK cells (110). Decidual T cells are predominantly of the CD8^+^ phenotype and play a role in the regulation of trophoblast invasion, while CD4^+^ Tregs promote tolerance to the fetus (105). Finally, DCs, even though occurring in very low numbers in the decidua, are believed to play a role in driving the differentiation of naïve CD4^+^ T cells into a Th2 phenotype and to regulate dNK proliferation and activation (105).

Regulation of immune responses through adequate activation of dNK cells, macrophages, and Tregs plays a role in the processes of implantation, placentation, and progression of pregnancy (75, 88). In the absence of immune regulation, continuous activation of monocyte phenotypes results in excessive immune activation that leads to a generalized inflammatory response (111). Regulatory products, cytokines, and other pro-inflammatory factors released from the placenta by syncytiotrophoblasts play a key role in signaling between cells of the inflammatory network (29, 109). Preeclampsia has been associated with disruption of many of these physiological processes. An ischemic placenta results in aberrant activation of immune cells by the continuous release of pro-inflammatory factors into the maternal circulation (111). Specifically, an imbalance in Th subsets, aberrant activation of dNK cells, and excessive recruitment of innate immune cells are mainly responsible for disrupting the regulation of immunological balance in pregnancy (111). These will now be discussed in turn.

### Imbalances Between Th Cell Subsets

The regulation of pro- and anti-inflammatory immune responses lies in the interplay between different Th cell subsets, especially between Th1 and Th2 cells, characterized by the release of pro-inflammatory (e.g., IL-2, IL-6, IL-8, IFN-γ, and TNF-α) and anti-inflammatory cytokines (IL-4, IL-10, and IL-13), respectively (112, 113). Th2 cytokines are involved in antibody production and take part in the regulation of inflammation in cooperation with IL-10-producing Tregs (88). During pregnancy, modulation of immune responses occurs, with distinct immunological features, observed at different stages (114). Implantation and placentation in the first and early second trimester of pregnancy are characterized by a pro-inflammatory environment that ensures adequate repair of the uterine epithelium and removal of cellular debris that accumulates secondary to embryo implantation (114). The second and third trimester are times of rapid fetal growth and development and the predominant immunological profile in the feto-maternal interface is anti-inflammatory (114). Progesterone, estradiol, prostaglandin D2, and leukemic inhibitory factor all promote the development of this Th2 profile (104, 105). The immunological effect of progesterone is mediated through a protein called P-induced blocking factor, which induces dominant Th2 cytokine production and immune modulation in aid of maternal tolerance toward the fetus (115). A Th2 bias in pregnancy modulates cell-mediated immunity, which is responsible for the increased susceptibility of pregnant women to bacterial LPS and intracellular pathogenesis of, for instance, *Listeria monocytogenes* (75, 104). Finally, during parturition, the immune profile reverts back to a pro-inflammatory state, which is necessary for the processes of delivery, such as contraction of the uterus and expulsion of the fetus and the placenta (104).

**Figure 2:**
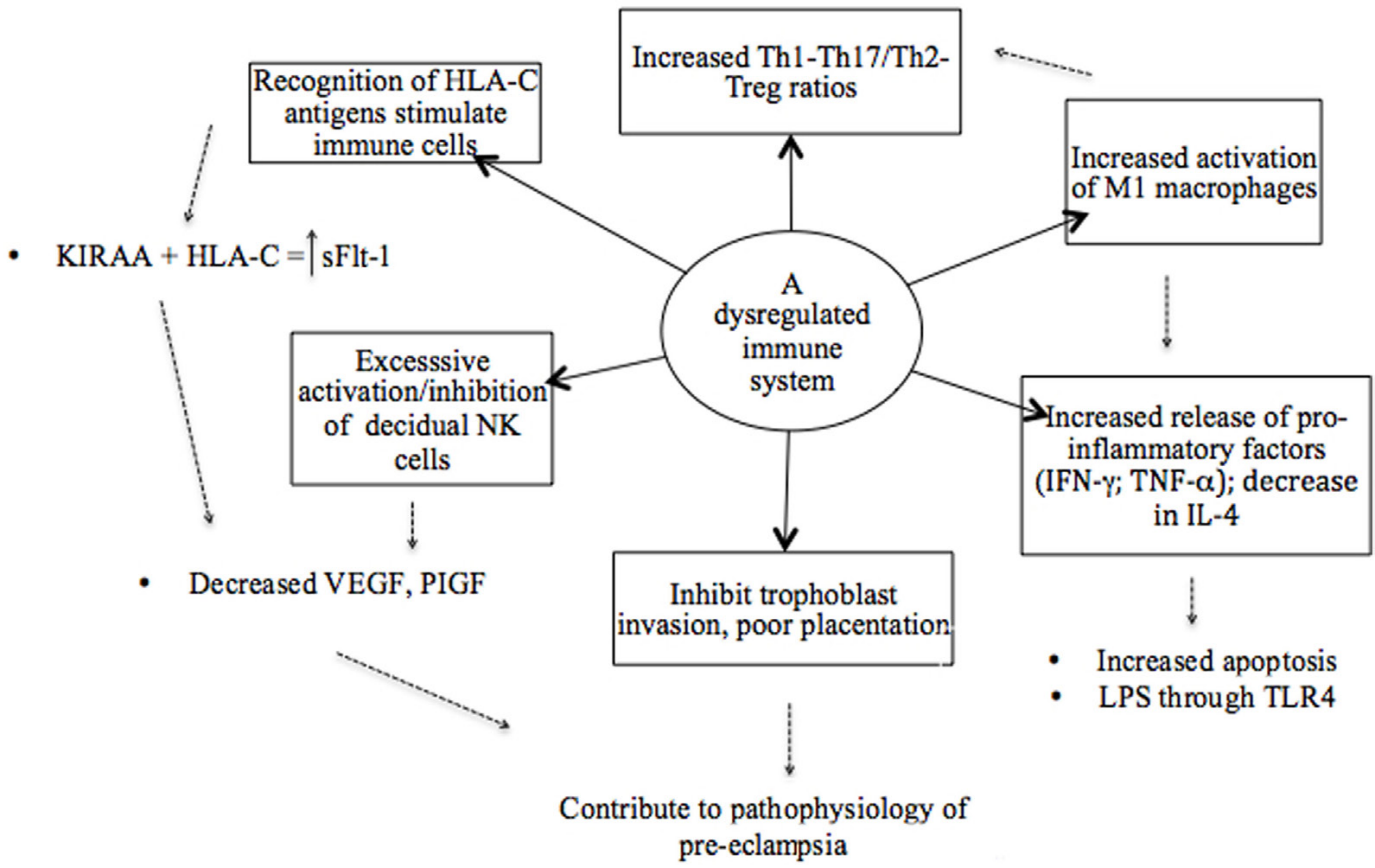
An illustrative model of the various components of a dysregulated immune system in preeclampsia. The recognition of antigens is considered a starting point where immune cells are stimulated based on the interaction between specific killer immunoglobulin-like receptor (KIR) and human leukocyte antigen (HLA) ligand genotypes. Subsequently, a dysregulated immune system in preeclampsia is attributed to an imbalance of pro- and anti-inflammatory cytokines as result of the overactivation of immune cells and pro-inflammatory stimuli that promote inflammation. Therefore, a hyperinflammatory environment in preeclampsia is established by the failure of regulatory mechanisms.

It has been proposed that a shift toward a Th2 profile in the second trimester does not occur in preeclampsia or, alternatively, that it is reversed in the early stages of the disease (71). This has the consequences that Th1 responses are not downregulated and cytokines exhibit mostly a pro-inflammatory profile, with elevated levels of IFN-γ and reduced levels of IL-4 and IL-10 reported (71, 116). Increased levels of Th1 type pro-inflammatory cytokines have also been associated with other pregnancy complications, such as preterm birth and IUGR (117). This pro-inflammatory systemic environment contributes to the failure of immune tolerance and results in immune dysregulation (Figure 2) (59, 118). The mediators that regulate this Th1/Th2 shift are not completely understood and are speculated to involve NK cells and IFN-γ, but also other cytokines, such as IL-12, IL-8, and the TGF-β family (117, 118). Interleukin-12 induces Th1 responses and stimulates the release of IFN-γ by NK and naïve T cells (7). In turn, IFN-γ primes monocytes to release more IL-12, increasing the production of IFN-γ. It has been suggested that this positive feedback cycle contributes to rapid deterioration in severe preeclampsia (7). Another key aspect in this paradigm is the negative feedback of IFN-γ on Th2 cells, in so doing suppressing immune regulation (71).

Possible mechanisms by which dominant Th1 immune responses induce the clinical aspects of preeclampsia are not completely known, but many of the respective Th1 cytokines (TNF-α, IL-2, IL-12, IL-18, and IFN-γ) have been reported to induce apoptosis of trophoblasts (25, 118). In addition, IL-12 and IFN-γ inhibit angiogenesis, whereas TNF-α activates endothelial cells and induces glomerular endothelial damage (7, 117). Tumor necrosis factor-α also inhibits the production of NO and thus influences the circulating levels of angiogenic factors in the second stage of preeclampsia (Figure 1) (118). Studies have, however, reported highly variable results of the circulating levels of TNF-α in preeclampsia, with some showing increased, unchanged, or even insignificantly decreased levels, possibly due to differences in timing and study method used (119–126). Nonetheless, serum TNF-α concentrations have been positively and significantly correlated with diastolic BP and uric acid levels, and it has hence been proposed as a potential marker of the severity of preeclampsia (125). TNF-α plays a critical role in maternal inflammation. Cotechini et al. (49) provided evidence to highlight the importance of this cytokine by using a mouse model where it was observed to induce abnormal inflammation in pregnant rats leading to FGR and features characteristic of preeclampsia (49). Elevated levels of IFN-γ are thought to play a central role in endothelial dysfunction and the exaggerated systemic inflammatory response in preeclampsia (Figure 2) (71). Overproduction of TNF-α and IFN-γ has also been reported in other adverse pregnancy outcomes, such as preterm birth (127) and IUGR, and could be suggested viewed as a primary target in preeclampsia pathology also because of their ability to inhibit sufficient angiogenesis, as previously mentioned (Figure 2) (117).

This one-dimensional Th1/Th2 paradigm can, however, not fully explain the immunological changes observed during preeclampsia and recent work has expanded the dichotomy to include the Th17 phenotype and a subset of CD4^+^ T cells known as CD25^+^ Forkhead box P3 gene (Foxp3^+^) Tregs (128). The latter are responsible for the development and regulation of immune responses and hence, the maintenance of immunological tolerance to the fetus (25, 112). Extrathymic and peripherally generated Tregs (pTregs) induce tolerance by suppressing the immune response generated by T-effector cells to foreign antigens, whereas thymic Tregs (tTregs) act to prevent autoimmunity by maintaining MHC class II antigen-specific tolerance (59).

Peripherally generated Tregs are produced within the decidualized endometrium during early pregnancy and are believed to play an essential role in pregnancy (129–131). Others have proposed that both tTregs and pTregs are indispensable, with tTregs potentially initiating a tolerant state and pTregs maintaining tolerance (132). Tregs have to be exposed to antigens presented by “tolerogenic” DCs in an appropriate cytokine environment for them to proliferate, mature, and eventually exert suppressive effects (128). Although HLA II is not expressed in either villous or extravillous cytotrophoblasts, antigenic stimulation is believed to come from the trophoblastic cell debris that contains intracellular fetal HLA-DR (59). It has also been suggested that Tregs recognize paternal HLA-C and can therefore downregulate anti-paternal responses, although the stability of this memory is still unknown and needs further investigation (79).

Regulatory T cells are differentiated according to the expression of surface markers, most notably the IL-2 receptor, CD25, and the transcription factor, forkhead box P3 (Foxp3^+^) (25, 59, 133). It has been suggested that the decidual CD4^+^ CD25^+^ Foxp3^+^ Tregs subsets specifically play a role in pregnancy by mediating immune tolerance to fetal antigens and suppressing inflammatory responses (134).

Regulatory T cells have many suppressive functions and targets, including suppression of activation, proliferation and cytokine release of CD4^+^ and CD8^+^ T cells, suppressing B-cell proliferation and immunoglobulin production, inhibiting the cytotoxic function of NK cells, and inhibiting maturation and function of antigen-presenting cells, such as DCs and macrophages (129). The molecular mechanisms by which Tregs exert their control are, however, incompletely understood. Some propose that their main action is through suppression of specific NK phenotypes and pro-inflammatory cytokines (71) or through the production of immunoregulatory cytokines, such as IL-10 and TGF-β that in turn promote and inhibit the differentiation of Tregs and Th17 cells, respectively (135, 136). Others believe the major mechanism to be through the expression of the membrane glycoprotein, cytotoxic T lymphocyte antigen (CTLA)-4 (137). The co-ligation of CTLA-4 with the T cell receptor results in increased expression of IDO, which effectively starves T cells by restricting the accessibility of the essential amino acid, tryptophan (137, 138). The interaction between Tregs and IDO is bidirectional, and the presence of IDO promotes the differentiation of T cells into Tregs, while in its absence, Tregs are reprogrammed to acquire a Th17 pro-inflammatory phenotype (79).

T-helper 17 cells are a CD4^+^ lymphocyte subpopulation, characterized by the production of the pro-inflammatory cytokine, IL-17 (112, 128). The Th17 cells induce a protective immune response against bacterial or fungal infections and the excessive upregulation of this subpopulation has been associated with the development and progression of autoimmune and chronic inflammatory diseases, allergies, and graft rejection (128, 139). A portion of IL-17A-producing cells have been found to also produce the Th1 cytokines, such as IFN-γ, and are hence termed Th17/Th1. Conversely, a small proportion of cells that have the ability to produce both IL-17A and IL-4 are termed Th17/Th2 cells. The latter clones originate in the presence of IL-4, suggesting that a microenvironment rich in IL-4 may induce a shift from Th17 to Th17/Th2 cells (140). IL-4 and HLA-G5 in the uterine environment is considered at least partly responsible for the development of specifically Th17/Th2 cells, which is crucial for successful implantation of the embryo in pregnancy (140, 141). Lombardelli et al. (141) reported that in successful pregnancy, a large number of CD4^+^ T cells produce both IL-17 and IL-4, whereas pathogenic decidual Th17/Th1 cells are commonly found in patients with unexplained recurrent abortion (141). The same study conducted by Lombardelli et al. (141) propose that the potentially detrimental effects of IL-17 may be counteracted when Th17 cells produce IL-17 together with IL-4 in the first trimester of pregnancy (141). IL-17/IL-4-producing CD4^+^ T cells promote an adequate response to extracellular pathogens, whereas IL-4 may induce tolerance toward the paternal HLA-C antigens and IL-17 could promote the proliferation and invasion of extravillous cytotrophoblasts (141).

A similar developmental lineage exists between Th17/Treg subsets, and the linked evolution of these subsets enables a balanced regulation of inflammation and autoimmunity (Figure 2) (84, 113, 142). Tregs and IL-17^+^ T cells are both involved in the establishment and maintenance of pregnancy, where these cells function as regulatory and effector cells, respectively (84, 143). Tregs act as regulators of Th17 cells and other immune cells involved in tolerance at the feto-maternal interfaces (25, 128).

Normal pregnancy is proposed to have a bias away from a Th17 response, while an increased ratio of IL-17^+^/Tregs has been demonstrated in cases of RPL, unexplained miscarriage, preterm birth, and preeclampsia (Figure 2) (59, 79, 113, 128, 139, 144). It has been shown that IL-17-producing lymphocytes are increased in the peripheral blood of preeclamptic patients in the third trimester of pregnancy and a significant correlation has been found between levels of Th17, IL-2-, and IFN-γ-producing T cells and the development of preeclampsia (145).

In preeclampsia, the uterine microenvironment acts as a contributing factor by influencing the differentiation of T cell subsets, especially Th17 cells (146). Therefore, further differentiation of Th17 cells could be related to the continuous release of pro-inflammatory cytokines and a dominant Th1 environment in preeclampsia (118, 146). The increased production of IL-1β and IL-6 by activated monocytes that induce an inflammatory microenvironment, as seen in recurrent abortions, can also participate in the expansion of Th17 and their conversion from Tregs (74, 147).

In addition to the regulatory action of Tregs, dNK cells are present in elevated levels in normal pregnancy and inhibit local inflammation at the feto-maternal interface and maintain tolerance by suppressing the development of pro-inflammatory Th17 cells (74, 136). The ratio of dNK/Th17 cells is decreased in cases of RPL abortion, indicating impaired regulatory activity and increased decidual inflammation (74). Deficient levels of Tregs and the absence of dNK cells lead to a prominent Th17 response, extensive local inflammation, and the failure of maternal–fetal tolerance, which could be possible mechanisms that play a role in the development of preeclampsia (74, 136).

Tolerance at the maternal–fetal interface is achieved through the production of the immunoregulatory cytokines, such as IL-10 and TGF-β1, that promote and inhibit the differentiation of Tregs and Th17 cells, respectively (136). Tregs also suppress innate and adaptive immune responses and play a major role in modulating the activity of self-reactive cells (112). The importance of Tregs in pregnancy was confirmed when the administration of these cells was found to prevent spontaneous abortion in pregnant mice (25). It is suggested that Tregs do not directly suppress Th1 responses in normal pregnancy, but regulate immune responses through the suppression of specific NK phenotypes and pro-inflammatory cytokine production (71). Although Tregs cannot reverse the shift toward a Th1 response by promoting Th2, these cells promote a tolerant microenvironment at the feto-maternal interface (148).

Preeclampsia has been associated with an imbalance in the two subpopulations of Tregs (25, 149). In comparison to normal pregnancy, preeclampsia is associated with increased numbers of CD4^+^CD25^high^Foxp3^+^ and decreased numbers and functional activity of CD4^+^CD25^+^Foxp3^high+^ cells (149). The stability of Tregs is suggested to be influenced by inflammatory environments, as has been demonstrated in inflammatory bowel disease (150). Pro-inflammatory cytokines, such as TNF-α, have been shown to have a direct negative effect on the function of Tregs, especially in cases of rheumatoid arthritis, and this may be an explanation for the altered levels of Tregs observed in preeclampsia (59, 150, 151). Furthermore, it has been suggested that a pro-inflammatory environment tends to suppress the generation of Tregs (79). In the pro-inflammatory environment of preeclampsia, IDO has been shown to be less active in late stages, possibly due to underactive Treg responses (79, 152). A decline in the level of decidual Tregs consequently leads to failed regulatory mechanisms, resulting in the immunological rejection of the fetus (25, 153).

### Aberrant Activation of Decidual NK Cells

In the first trimester of pregnancy, a subset of peripheral blood NK cells (decidual CD16^−^CD56^bright^ NK cells—dNK cells) differentiates to constitute the major immune cell population in the decidua (59, 75). Peripheral NK cells (CD16^+^CD56^dim^) are cytotoxic, express high levels of KIRs and CD57 (a marker of cell maturation and potent cytotoxic potential). and do not secrete cytokines (154, 155). By contrast, CD16^−^CD56^bright^ cells do not induce antibody-mediated cell toxicity and express only low levels of perforin, but high levels of the inhibitory receptor CD94/NKG2 (154). Although CD16^−^CD56^bright^ NK cells in the decidua possess cytotoxic properties, these cells are an important source of immunoregulatory cytokines, such as IL-10 and TGF-β1, which play an important role in tolerance and successful pregnancy by directly inhibiting the proliferation of Th17 cells and suppressing inflammatory responses, as mentioned earlier (7, 25, 74, 81, 87, 136). While dNK cells also secrete their classic, pro-inflammatory cytokine, IFN-γ, its effect is mainly exerted through inducing spiral artery remodeling by initiating vessel instability (74, 156). The secretion of chemokines, IL-8, and the IFN-inducible-protein (IP)-10 by dNK cells facilitates the migration of the extravillous cytotrophoblast into the *decidua basalis* and results in the invasion of spiral arteries, further contributing to uterine vascular remodeling crucial for placental development (87).

The phenotype of dNK cells can be altered by severe inflammation in the uterine microenvironment caused by severe stress, viral infection, and autoimmune diseases (74). The expression of the marker CD27 on NK cells, which correlates with an increased ability to proliferate and produce IFN-γ and with lower cytotoxic potential (157), has been suggested to be an important marker in distinguishing different subsets of NK cells (74). A large number of CD27^+^ NK cells have been found in the decidua, where this subset of NK cells is an important source of cytokines and shows limited cytotoxicity (74). Studies of RPL as a model pathogenic state demonstrated an altered ratio of CD56^bright^ CD27^+^ to Th17 cells, impaired release of the immunoregulatory cytokines, such as IL-10 and IFN-γ, as well as the failed inhibition of inflammation (74, 158). A possible explanation for these findings is that dNK cells may have increased cytotoxicity toward inflammatory cells together with impaired regulatory capacity, and that prolonged overactivation of dNK cells may result in them becoming immunodepleted and adopting an abnormal phenotype (74).

Preeclampsia is associated with a shift in NK cells toward a cytotoxic phenotype with increased production of IFN-γ, which causes apoptosis of cytotrophoblasts, thus inhibiting trophoblast invasion (25, 159). In addition, the lower expression of the natural cytotoxicity receptor, NKp46, on NK cells in women with preeclampsia has been suggested to increase the production of the pro-inflammatory cytokine, TNF-α, which further drives a shift in NK cells (160). Accordingly, inappropriate recruitment of peripheral blood NK cells to specialized decidual cells may lead to the development of a cytotoxic environment *in utero* (113). Hence, successful implantation and placentation depend on a fine balance between decidual NK cell infiltration and activation of regulatory phenotypes (113).

### Excessive Recruitment of Innate Immune Cells

In preeclampsia, monocytes, neutrophils, and macrophages are continuously activated due to the exposure to elevated levels of pro-inflammatory factors in the uterine microenvironment (109). Monocytes invade tissues upon an inflammatory stimulus and subsequently develop into macrophages that produce factors associated with tissue remodeling and angiogenesis in pregnancy (109, 161). In the decidua, macrophages are broadly distinguished into two phenotypes: (i) M1 macrophages with an inflammatory and microbicidal nature and (ii) M2 macrophages with immunosuppressive properties that maintain immunological homeostasis during pregnancy (75). The latter phenotype mainly represents decidual macrophages in pregnancy and contributes to immune regulation by producing immunosuppressive cytokines, such as IL-10 and TGF-β1, to regulate inflammation and suppress Th1 cell polarization (7, 75, 88). Decidual macrophages secrete angiogenic factors to promote placentation and the regulatory properties of these macrophages enable them to remove apoptotic cells to prevent the harmful effects of these pro-inflammatory factors (75, 127, 161). The decrease of immunosuppressive cytokines observed in preeclamptic patients indicates altered M2 macrophage polarization as reduced M2 numbers in the decidua contribute to failure of immune regulation (75, 162). This is expected because the polarization of M2 macrophages relies on exposure of type 2 immunosuppressive cytokines such as IL-10 that is present in low concentrations in preeclampsia (161).

When immune regulatory mechanisms are not intact, augmented inflammation will induce M1 macrophages and apoptosis of cytotrophoblasts, contributing to poor placentation (88). Increased apoptosis of cytotrophoblasts and syncytiotrophoblasts is attributed to elevated levels of pro-inflammatory TNF-α and IFN-γ that inhibit trophoblast invasion and spiral artery remodeling (127, 163, 164). In preeclampsia, excessive inflammation or oxidative stress in the placenta leads to increased cell death of syncytiotrophoblasts (7, 19). Increased placental apoptotic debris in preeclampsia is suggested to participate in the pathogenesis of the disorder by enhancing the inflammatory stimulus with or without specific immune recognition (7). The phagocytosis of these large numbers of necrotic or apo-necrotic trophoblasts by macrophages and neutrophils may lead to increased production of pro-inflammatory type 1 cytokines, such as TNF-α, IL-12, and IFN-γ (7, 117). Excessive apoptosis creates a danger signal and activates macrophages toward a pro-inflammatory cytokine profile (7). Furthermore, the activation of M1 macrophages by pro-inflammatory stimuli (TNF-α, IFN-γ, and bacterial LPS) inhibits trophoblast invasion that further contributes to the imbalance between Th1 and Th2 cytokines and the exaggerated inflammatory response observed in preeclampsia (Figure 2) (59, 127).

## The Impact of a Disrupted Immune System on the Metabolic Changes Associated with Preeclampsia

In preeclampsia, a dysregulated immune system is a result of dominant Th1 subsets, the elevated release of pro-inflammatory cytokines from the placenta, aberrant activation of macrophages and dNK phenotypes that continuously promote a pro-inflammatory environment, which further activates other immune cells (26). Elevated levels of inflammatory cytokines, specifically TNF-α and IL-6, generate widespread dysfunction of the maternal vascular endothelium that could result in hypertension (17). Increased pro-inflammatory cytokines also affect metabolic changes in preeclampsia. For instance, elevated levels of TNF-α induce insulin resistance and stimulate the adipocytes to release more free fatty acids (FFAs) (21, 165). Circulating levels of FFAs are increased in preeclampsia, contributing to insulin resistance and altered lipid metabolism in this disorder (165, 166). Several metabolic disorders are also associated with systemic inflammation and, as discussed previously, there is pathological resemblance between preeclampsia and the components of the metabolic syndrome (21). Obesity is a metabolic disorder characterized by systemic inflammation and an increased risk of developing preeclampsia. In obese individuals, adipose tissue is suggested to be a potent source of pro-inflammatory stimuli because of the ability of adipocytes to secrete the pro-inflammatory cytokines, such as TNF-α and IL-6 (21). Besides promoting inflammation, these cytokines also advance atherogenesis, the underlying pathological process in atherosclerosis (167). Atherosclerosis is an inflammatory disorder associated with endothelial dysfunction, similar to preeclampsia, and supports the hypothesis that a disrupted immune system, specifically a pro-inflammatory bias, is associated with metabolic disorders (167).

Obese individuals thus have increased susceptibility to preeclampsia during pregnancy because of an already increased inflammatory response (21). Interestingly, endotoxin (bacterial LPS) of Gram-negative bacteria such as *Escherichia coli* has been shown to trigger and maintain a low-grade inflammatory state (168). In a mouse model, injection of LPS induced increased expression of pro-inflammatory cytokines, such as TNF-α, monocyte chemoattractant protein-1, and IL-6 (169). In addition, endotoxin stimulates the expression of IL-1 and TNF-α in endothelial- and vascular smooth muscle cells (167, 170). IL-1 causes alterations in endothelial function, such as the induction of procoagulant activity that promotes blood clotting, that may play an important role in the vascular effects of inflammation and the pathogenesis of vascular diseases (170).

Innate recognition of LPS occurs through a receptor complex consisting of CD14, TLR-4, and myeloid differentiation factor 2 that recognizes pathogenic structures and subsequently induces an inflammatory response. Continuous activation of the innate immune system through TLRs by bacterial stimuli may disrupt immune homeostasis by the overproduction of pro-inflammatory cytokines that can lead to a systemic inflammatory response (171). In preeclampsia, the increased expression of TLR-4 by placental trophoblasts increases the secretion of trophoblast chemokines, thus attracting more monocytes to the decidua (59). The increased recruitment of innate immune cells and subsequent increased expression of inflammatory mediators at the fetal–maternal interface caused by bacterial stimuli can also trigger preterm birth (172).

Previous studies suggested TLR-4 activation to be associated with inflammation in preeclampsia (173–175). Supporting this suggestion, a later study found a direct correlation between TLR-4 activation, inflammation, and plasma and placental oxidative damage in preeclampsia (176). These results suggest that the upregulation of TLR-4 activation in preeclampsia could be contributing to the systemic inflammatory response following local and systemic oxidative damage (176). Therefore, through the activation of TLR-4, bacterial stimuli may contribute to the oxidative, inflammatory, and metabolic stresses in preeclampsia (21).

In this review, we strongly support the role of a dysregulated immune system in preeclampsia. Pro-inflammatory stimuli may be responsible for disrupting immune homeostasis in this disorder and we therefore suggest that preexisting systemic inflammation, likely caused by multiple factors, including bacterial endotoxin, to cause preeclampsia. In addition to increased pro-inflammatory factors released by the placenta and activated immune cells contributing to excessive systemic inflammation, we suggest that bacterial endotoxin could also induce inflammatory responses as a predisposing factor to the oxidative damage and systemic inflammation in preeclampsia.

## Conclusion

The precise mechanisms operative in the development of preeclampsia are unknown; however, evidence suggests that chronic inflammation, inadequate invasion of trophoblasts, poor angiogenesis, and the failure of immunological tolerance are co-influencing factors in the placental subtype of this disorder (146). Dysregulation of the immune system during pregnancy can lead to abnormal activation of innate immune responses that result in pregnancy complications, such as preeclampsia and IUGR (75). In pregnancy, emphasis is on the direct and indirect control of inflammation to maintain tolerance at the fetal–maternal interface, while still protecting the host. Trophoblast invasion, angiogenesis, and placentation are regulated by the secretion of angiogenesis-regulating molecules, chemokines and cytokines by phenotypically activated NK cells, T cell subsets, and macrophages (81). The unbalanced activation of the main immune mediators causes disruption of the regulation of immune mechanisms contributing to the failure of immune tolerance by inducing a cytotoxic environment that leads to endothelial dysfunction (17). However, in addition to the pro-inflammatory factors of the placenta, bacterial endotoxin could also act as a stimulus to disrupt the pregnancy-specific immune balance and cause the pathological events of preeclampsia. Future research needs to investigate the role of infectious agents as alternatives to placental factors to be responsible for the disruption of the immune system in preeclampsia.

## Author Contributions

JG was the project leader and involved in the writing of the manuscript. TR, HL, MK, and ME were involved in the review of the manuscript. All the authors read and approved the manuscript.

## Conflict of Interest Statement

The authors declare that the research was conducted in the absence of any commercial or financial relationships that could be construed as a potential conflict of interest.
